# Effect of a Magnetic Field on Drosophila under Supercooled Conditions

**DOI:** 10.1371/journal.pone.0051902

**Published:** 2012-12-28

**Authors:** Munekazu Naito, Shuichi Hirai, Makoto Mihara, Hayato Terayama, Naoyuki Hatayama, Shogo Hayashi, Masayuki Matsushita, Masahiro Itoh

**Affiliations:** 1 Department of Anatomy, Tokyo Medical University, Tokyo, Japan; 2 Department of Plastic and Reconstructive Surgery, Graduate School of Medicine, University of Tokyo, Tokyo, Japan; 3 Medicinal Education Center, School of Medicine, Aichi Medical University, Aichi, Japan; 4 Molecular and Cellular Physiology, University of the Ryukyus, Okinawa, Japan; Alexander Flemming Biomedical Sciences Research Center, Greece

## Abstract

Under subzero degree conditions, free water contained in biological cells tends to freeze and then most living things die due to low temperatures. We examined the effect of a variable magnetic field on Drosophila under supercooled conditions (a state in which freezing is not caused even below the freezing point). Under such supercooled conditions with the magnetic field at 0°C for 72 hours, −4°C for 24 hours and −8°C for 1 hour, the Drosophila all survived, while all conversely died under the supercooled conditions without the magnetic field. This result indicates a possibility that the magnetic field can reduce cell damage caused due to low temperatures in living things.

## Introduction

Generally, water changes from liquid to solid at 0°C. However, when the temperature of water is gradually lowered using a program freezer, etc., a “supercooling phenomenon” occurs in which the water does not change phase from liquid to solid even when the temperature falls below 0°C. Furthermore, it has been clarified that this supercooling phenomenon is stably caused by artificially applying pressure and voltage to water [1]–[3]. We recently invented a program freezer that causes supercooling conditions using a magnetic field. It is known that when the temperature is further reduced from the supercooled condition using this device in order to freeze the internal organs, the shape of the tissues maintains a better form compared to the group that used liquid nitrogen [4]. In physical experiments, it has been clarified that uniform ice crystals with small cracks are formed when a variable magnetic field is added to the coagulation process of water, thus suggesting that this caused a reduction in damage to tissues [5]. However, the detailed mechanism of this action has not yet been clarified.

On the other hand, it is known that the metabolism of organisms declines when low temperatures are reached. In theory, when a cell is frozen at very low temperatures, the metabolic activities of the cells stop, thus allowing for the semipermanent maintenance of the state before freezing. To date, cryopreservation of cells such as sperm [6], red blood cells [7], etc. has become possible by adding glycerol, etc. Moreover, regarding organs, cryopreservation has been successfully achieved in mouse and pig livers [8] as well as in human ovaries [9]. However, the cryopreservation of an organism is difficult at this point due to severe tissue damage following thawing.

It is becoming clear that in humans, therapeutic hypothermia increases the resuscitation rate following cardiopulmonary arrest [10]. In addition, in the natural world, several organisms live or hibernate by controlling their metabolism to below 0°C. Frogs can live at −6°C with glucose as antifreeze [11], fish inhabiting Antarctica with antifreezing protein can live at −2°C [12], and arctic squirrels can live at −2.9°C by bringing their internal bodies to a supercooled condition [13]. Therefore, in this study, we prepared a supercooled condition (a state in which freezing is not caused even below the freezing point) using a program freezer and examined how a magnetic field affects organisms using Drosophila.

## Materials and Methods

### *Drosophila*

Drosophila adult flies (Oregon-R: wild type) were purchased from the Kagaku club (Shiga, Japan) and they were raised on a standard cornmeal medium at 25°C.

### Program freezer with a variable magnetic field

**Figure 1:**
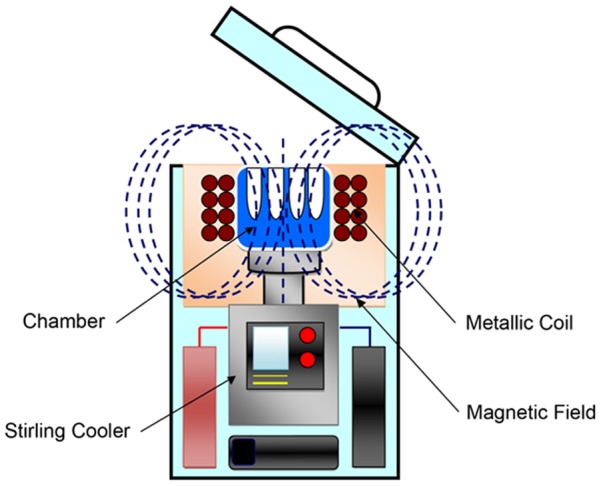
System of program freezer using magnetic field. Function: Cooling while generating a 0.5 mT magnetic field inside the chamber is possible by electrifying the coil from the generator.

An aluminum cooling chamber (inner diameter: 125.5×93 mm; depth: 40 mm) was installed in the cooling unit of a stirling engine, which is a cooling device, and a hole with an inner diameter of 12.8 mm and depth of 32 mm was made inside to insert the specimens. The chamber was covered with a heat insulating material, with a teflon-coated coil of wire coiled around the outside thereof. Alternating current was applied using a specialized function generator, the coil was electrified with 30 Hz, 2 V, 0.8 A, and a variable magnetic field of 0.5 mT (tesla) was caused inside the chamber. The entire device is shown in Fig. 1.

### Evaluation of the cooling process on distillated water and saline with or without a magnetic field

1 ml distilled water (freezing point: 0°C) and 1 ml saline (freezing point: −0.65°C) were put in a plastic tube (Nunc cryotube, Thermo Fisher Scientific Inc, Waltham, USA), a magnetic field with an intensity of 0.5 mT was added, and it was cooled at a regular speed of −3°C/min. Those without a magnetic field added were classified as the control group. A type K thermocouple sensor was installed inside the chamber, and the temperature and the time were measured using a thermometer (PC card-type data acquisition system, type: NR-250, KEYENCE CORPORATION “Keyence”, Japan, Osaka). To clarify whether a variable magnetic filed affects the temperature, the experiments were repeated fifteen times, respectively, and the data were analyzed by the non-paired *t*-test and F-test for the time required to reach 0, −2, −4, −6, −8 and −10°C.

### Supercooling preservation experiment on Drosophila with or without a magnetic field

Five to ten Drosophila were put into 2.5 ml plastic tubes respectively. This tube was set in a chamber and it was cooled from room temperature at −3°C/min. The point at which 5 minutes elapsed after the target temperature had been reached was determined to be 0 hours. The Drosophila were preserved at 0°C for 1 hour, 6 hours, 12 hours, 24 hours, 48 hours, 72 hours and 96 hours; at −2 and −4°C for 1 hour, 6 hours, 12 hours, 24 hours and 48 hours; at −6 and −8°C for 1 hour and 6 hours; and at −10°C for 1 hour. The experimental group was exposed to a variable magnetic field with an intensity of 0.5 mT from the beginning of the cooling. Subsequently, the tube was taken from the freezer and re-warmed to room temperature. The Drosophila were observed for 24 hours to check for survival. Those that were not exposed to a variable magnetic field added were classified as the control group. To clarify whether the survival rate of Drosophila is related to a magnetic filed, the data were analyzed using a three-parameter logistic regression model of the survival rate by time, temperature and with/without a variable magnetic field.

## Results

**Figure 2:**
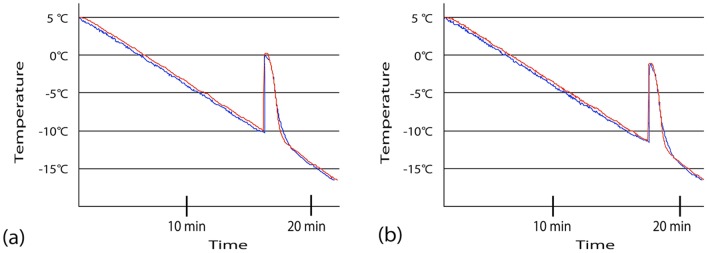
Supercooling phenomenon in distilled water (**a**) **and normal saline** (**b**) **under magnetic field** (**Red line**) **or not** (**Blue line**)**.** Each line shows the average temperature of fifteen experiments every 10 second. Supercooling is an unstable phenomenon in which the temperature is brought below the freezing point but a phase change from liquid to solid does not occur.

The temperature change of distilled water and saline is shown in Fig. 2. When distilled water was put in a tube, the temperature gradually dropped by 3°C per minute from a room temperature of 20°C, and the supercooled condition continued until −10°C was reached (Fig. 2a). When saline was put in a tube, the temperature gradually dropped by 3°C per minute in the same manner as when distilled water was put in; however, the supercooled condition continued until −12°C was reached. In both distilled water and saline, the time to reach 0, −2, −4, −6, −8 or −10°C were equivalent in averages and variables weather a magnetic field was added or not.

Next, the outcome of preserving Drosophila is shown in Table 1. When Drosophila were preserved for 72 hours at 0°C, 13 of 60 survived in the experimental group while all 47 Drosophila died in the control group. Under conditions at −2 and −4°C for 24 hours, 25 of 54 and 15 of 45 survived in the experimental group, respectively, while all 106 Drosophila died in the control group. Under the condition at −8°C for 1 hour, 57 of 67 survived in experimental group, while in the control group, all 66 Drosophila died. Under a condition of −10°C, all 48 Drosophila died following 1 hour of preservation for both groups. When the Drosophila were observed under a microscope immediately following preservation under a supercooled condition of −2°C or less, there was no body movement at all. However, the surviving Drosophila gradually commenced body movement with re-warming and then widely fluttered about several hours later.

**Table 1:** The survival rate of the *Drosophila* at 24 hours after the preservation in the supercooled condition with or without the magnetic field.

	Time						
Temperature	1h	6h	12h	24h	48h	72h	96h
0°C	57/57 (60/60)	60/60 (39/39)	63/72 (54/60)	3/60 (54/64)	15/63 (3/63)	13/60 (0/47)	0/48 (0/36)
−2°C	45/45 (48/48)	44/44 (45/45)	62/68 (12/73)	25/54 (0/49)	0/43 (0/42)	−	−
−4°C	39/39 (44/46)	63/63 (44/64)	57/64 (3/60)	15/45 (0/57)	0/49 (0/39)	−	−
−6°C	68/68 (28/44)	0/62 (0/52)	−	−	−	−	
−8°C	57/67 (0/66)	0/55 (0/50)	−	−	−	−	−
−10°C	0/49 (0/48)	−	−	−	−	−	−

The parentheses indicate the condition without the magnetic filed.

In a logistic regression model, the survival rate was significantly affected by time (chi square = 904.4, p<0.001), temperature (chi square = 645.2, p<0.001) and weather magnetic field are added or not (chi square = 224.5, p<0.001) respectively. Especially, the odds ratio associated with the magnetic field was 12.6, indicating that Drosophila in a magnetic field had 12.6 times higher survival than without a magnetic field. The interactions between them were also significant (p<0.001) but much smaller (chi-square  = 27.3–67.2).

## Discussion

In this experiment, it was shown that Drosophila survive when they are preserved at 0°C for 72 hours, at −4°C for 24 hours, and at −8°C for 1 hour under a supercooled condition with a 0.5 mT variable magnetic field added. As far as we know, this is the first report investigating how magnetic fields affect organisms under supercooled conditions.

In previous reports, several experiments examined whether or not a supercooled condition is useful for preserving rat organs [1]–[3]. Takahashi et al. [1] reported that when livers were preserved under a supercooled condition of −2°C with 5MPa pressure added, the shape of the tissue was maintained and the success rate of liver transplantation improved. Monzen et al. [2] clarified that when the heart, liver and kidneys are preserved under a supercooled condition of −4°C with 100 Volt and 500 Volt added, the biomarker of organ preservation declines compared to preservation at 4°C. Moreover, Okamoto et al. [3] reported that when the lungs are preserved under a supercooled condition of −2°C with 3000 volt added, reperfusion injury clearly declines compared to when preserving at 4°C. In these articles, the authors conclude that the supercooled condition lowered the metabolism of cells and decreased cell damage. In our experiment, when Drosophila were preserved under supercooled conditions at 0°C for 72 hours, −4°C for 24 hours and −8°C for 1 hour without a magnetic filed, the Drosophila all died. Perhaps while the metabolism of the cells declined when low temperatures were reached, cell damage was caused due to the low temperatures. However, when Drosophila were preserved under supercooled conditions with a 0.5 mT magnetic field added, the some Drosophila survived. Especially, fifty-seven of 67 Drosophila exposed to a magnetic field at −8°C for 1 hour, survived, while all of the Drosophila in the control group died. It is well known that cold injuries cause mortality at much higher temperatures, typically around −5°C [14], [15]. Our results suggest that the magnetic field reduced the cell damage induced by low temperatures. Recently, it was reported that either by direct effect on paramagnetic ROS (reactive oxygen species) or indirect changes to signaling pathways the magnetic field might affect the oxidative stress of reperfusion injury [16]. Moreover, electron paramagnetic resonance provides a non-invasive way to assay ROS build up during preservation if titration or the like are unappealing [17]. Therefore, ROS may be one of the important factors to explain the mechanisms of effects of magnetic filed on Drosophila.

In 1979, it was reported in the United States of America that leukemia frequently occurs in residents living near high voltage lines, and since then, experiments regarding the effect of magnetic fields on organisms have been strenuously advanced [18]. For example, in experiments using rats, it was reported that exposure to a 0.1 mT magnetic field promoted the onset of mammary gland tumors and ornithine decarboxylase activation [19]. Moreover, it is reported that when a 200 to 400 mT magnetic field was exposed to cells originating from rat pheochromocytoma (PC12-VG), change was observed in intracellular signaling, such as an influx of ions via the cell membrane [20]. Meanwhile, Bassett et al. [21] reported that a 0.2 mT magnetic field was effective for healing bone fractures in human and Oshibuchi [22] reported that a 10 mT magnetic field was effective for healing wounds in mice. However, many reports disproving these effects of magnetic fields have also been published and no uniform view has yet been reached [23]. Regarding the reasons thereof, the fact that no biological indicator showing high sensitivity in the magnetic field has yet been identified. Moreover, in experiments using magnetic fields, it is difficult to separate the effect of the magnetic field itself and the stimulation and/or heating effect on the excitable tissue, so problems with the experiments themselves have also been suggested [23]. In this experiment, a 0.5 mT magnetic field did not affect the transition of the temperature of distilled water or saline. Indeed it remains unclear whether the Drosophila are frozen or not under these conditions, but it is certain that a very weak magnetic field affects the life or death of such Drosophila under supercooled conditions. The present study involved a single condition of magnetic field (30 Hz, 0.5 mT). In the future, we plan to study the effects of other frequencies and intensities of magnetic fields on organisms under supercooled conditions.

Recently, Cho et al. [24] developed magnetic nanoparticles that turn on apoptosis cell signaling using a magnetic field in a remote and non-invasive manner. They also demonstrated that the observed magnetic switch operates on the micrometre scale and it can also be applied to an in vivo system where apoptotic morphological changes of Zebrafish can be successfully induced. These techniques may therefore help to elucidate the mechanism of a magnetic field on Drosophila under supercooled conditions.
